# A comparison of classification methods for predicting Chronic Fatigue Syndrome based on genetic data

**DOI:** 10.1186/1479-5876-7-81

**Published:** 2009-09-22

**Authors:** Lung-Cheng Huang, Sen-Yen Hsu, Eugene Lin

**Affiliations:** 1 Department of Psychiatry, National Taiwan University Hospital Yun-Lin Branch, Taiwan; 2 Graduate Institute of Medicine, Kaohsiung Medical University, Kaohsiung, Taiwan; 3 Department of Psychiatry, Chi Mei Medical Center, Liouying, Tainan, Taiwan; 4 Vita Genomics, Inc, 7 Fl, No 6, Sec 1, Jung-Shing Road, Wugu Shiang, Taipei, Taiwan

## Abstract

**Background:**

In the studies of genomics, it is essential to select a small number of genes that are more significant than the others for the association studies of disease susceptibility. In this work, our goal was to compare computational tools with and without feature selection for predicting chronic fatigue syndrome (CFS) using genetic factors such as single nucleotide polymorphisms (SNPs).

**Methods:**

We employed the dataset that was original to the previous study by the CDC Chronic Fatigue Syndrome Research Group. To uncover relationships between CFS and SNPs, we applied three classification algorithms including naive Bayes, the support vector machine algorithm, and the C4.5 decision tree algorithm. Furthermore, we utilized feature selection methods to identify a subset of influential SNPs. One was the hybrid feature selection approach combining the chi-squared and information-gain methods. The other was the wrapper-based feature selection method.

**Results:**

The naive Bayes model with the wrapper-based approach performed maximally among predictive models to infer the disease susceptibility dealing with the complex relationship between CFS and SNPs.

**Conclusion:**

We demonstrated that our approach is a promising method to assess the associations between CFS and SNPs.

## Background

Chronic fatigue syndrome (CFS) affects at least 3% of the population, with women being at higher risk than men [1]. CFS is characterized by at least 6 months of persistent fatigue resulting in substantial reduction in the person's level of activity [2-4]. Furthermore, in CFS, four or more of the following symptoms are present for 6 months or more: unusual post exertional fatigue, impaired memory or concentration, unrefreshing sleep, headaches, muscle pain, joint pain, sore throat and tender cervical nodes [2-4]. It has been suggested that CFS is a heterogeneous disorder with a complex and multifactorial aetiology [3]. Among hypotheses on aetiological aspects of CFS, one possible cause of CFS is genetic predisposition [5].

Single nucleotide polymorphisms (SNPs) can be used in clinical association studies to determine the contribution of genes to disease susceptibility or drug efficacy [6,7]. It has been reported that subjects with CFS were distinguished by SNP markers in candidate genes that were involved in hypothalamic-pituitary-adrenal (HPA) axis function and neurotransmitter systems, including catechol-O-methyltransferase (COMT), 5-hydroxytryptamine receptor 2A (HTR2A), monoamine oxidase A (MAOA), monoamine oxidase B (MAOB), nuclear receptor subfamily 3; group C, member 1 glucocorticoid receptor (NR3C1), proopiomelanocortin (POMC) and tryptophan hydroxylase 2 (TPH2) genes [8-11]. In addition, it has been shown that SNP markers in these candidate genes could predict whether a person has CFS using an enumerative search method and the support vector machine (SVM) algorithm [9]. Moreover, the gene-gene and gene-environment interactions in these candidate genes have been assessed using the odds ratio based multifactor dimensionality reduction method [12] and the stochastic search variable selection method [13].

In the studies of genomics, the problem of identifying significant genes remains a challenge for researchers [14]. Exhaustive computation over the model space is infeasible if the model space is very large, as there are 2^p ^models with p SNPs. [15]. By using feature selection techniques, the key goal is to find responsible genes and SNPs for certain diseases or certain drug efficacy. It is vital to select a small number of SNPs that are significantly more influential than the others and ignoring the SNPs of lesser significance, thereby allowing researchers to focus on the most promising candidate genes and SNPs for diagnostics and therapeutics [16,17].

The previous findings [8,9] mainly reported of modeling disease susceptibility in CFS by using machine learning approaches without feature selection. In this work, we extended the previous research to uncover relationships between CFS and SNPs and compared a variety of machine learning techniques including naive Bayes, the SVM algorithm, and the C4.5 decision tree algorithm. Furthermore, we employed feature selection methods to identify a subset of SNPs that have predictive power in distinguishing CFS patients from controls.

## Materials and methods

### Subjects

The dataset, including SNPs, age, gender, and race, was original to the previous study by the CDC Chronic Fatigue Syndrome Research Group [18]. More information is available on the website [18]. In the entire data set, there were 109 subjects, including 55 subjects having had experienced chronic fatigue syndrome (CFS) and 54 non-fatigued controls. Table 1 demonstrates the demographic characteristics of study subjects.

**Table 1:** Demographic information of study subjects.

**Factor**	**Subjects**
CFS/non-fatigue (n)	55/54
Age (year)	50.5 ± 8.5
Male/Female (n)	16/93
Race; white/black/other (n)	104/4/1

CFS = chronic fatigue syndrome.

Data are presented as mean ± standard deviation.

### Candidate genes

In the present study, we only focused on the 42 SNPs as described in Table 2[18]. As shown in Table 2[18], there were ten candidate genes including COMT, corticotropin releasing hormone receptor 1 (CRHR1), corticotropin releasing hormone receptor 2 (CRHR2), MAOA, MAOB, NR3C1, POMC, solute carrier family 6 member 4 (SLC6A4), tyrosine hydroxylase (TH), and TPH2 genes. Six of the genes (COMT, MAOA, MAOB, SLC6A4, TH, and TPH2) play a role in the neurotransmission system [8]. The remaining four genes (CRHR1, CRHR2, NR3C1, and POMC) are involved in the neuroendocrine system [8]. The rationale of selecting these SNPs is described in detail elsewhere [8]. Briefly, most of these SNPs are intronic or intergenic except that rs4633 (COMT), rs1801291 (MAOA), and rs6196 (NR3C1) are synonymous coding changes [8].

**Table 2:** A panel of 42 SNPs by the CDC Chronic Fatigue Syndrome Research Group.

**Gene**	**SNPs**
COMT	rs4646312, rs740603, rs6269, rs4633, rs165722, rs933271, rs5993882
CRHR1	rs110402, rs1396862, rs242940, rs173365, rs242924, rs7209436
CRHR2	rs2267710, rs2267714, rs2284217
MAOA	rs1801291, rs979606, rs979605
MAOB	rs3027452, rs2283729, rs1799836
NR3C1	rs2918419, rs1866388, rs860458, rs852977, rs6196, rs6188, rs258750
POMC	rs12473543
SLC6A4	rs2066713, rs4325622, rs140701
TH	rs4074905, rs2070762
TPH2	rs2171363, rs4760816, rs4760750, rs1386486, rs1487280, rs1872824, rs10784941

The "rs number" means the NCBI SNP ID.

COMT = catechol-O-methyltransferase, CRHR1 = corticotropin releasing hormone receptor 1, CRHR2 = corticotropin releasing hormone receptor 2, MAOA = monoamine oxidase A, MAOB = monoamine oxidase B, NR3C1 = nuclear receptor subfamily 3, group C, member 1 glucocorticoid receptor, POMC = proopiomelanocortin, SLC6A4 = solute carrier family 6 member 4, SNP = Single nucleotide polymorphism, TH = tyrosine hydroxylase, TPH2 = tryptophan hydroxylase 2.

In this study, we imputed missing values for subjects with any missing SNP data by replacing them with the modes from the data [19]. In the entire dataset, 1.08% of SNP calls were missing. Because there are three genotypes per locus, each SNP was coded as 0 for homozygote of the major allele, 1 for heterozygote, and 2 for homozygote of the minor allele, respectively.

### Classification algorithms

In this study, we used three families of classification algorithms, including naive Bayes, SVM, and C4.5 decision tree, as a basis for comparisons. These classifiers were performed using the Waikato Environment for Knowledge Analysis (WEKA) software [19]. First, naive Bayes is the simplest form of Bayesian network, in which all features are assumed to be conditionally independent [20]. Let (X_1_,..., X_p_) be features (that is, SNPs) used to predict class C (that is, disease status, "CFS" or "control"). Given a data instance with genotype (x_1_,..., x_p_), the best prediction of the disease class is given by class c which maximizes the conditional probability Pr(C = c | X_1 _= x_1_,..., X_p _= x_p_). Bayes' theorem is used to estimate the conditional probability Pr(C = c | X_1 _= x_1_,..., X_p _= x_p_), which is decomposed into a product of conditional probabilities.

Second, the SVM algorithm [21], a popular technique for pattern recognition and classification, was utilized to model disease susceptibility in CFS with training and testing based on the smaller dataset. Given a training set of instance-label pairs (*x*_*i*_, *y*_*i*_), *i *= 1,..., *n*, the SVM algorithm solves the following optimization problem [21]:



where **x**_*i *_∈ *R*^N ^are training vectors in two classes, ***y ***∈ *R*^*n *^is a vector such that *y*_*i *_∈ {1, -1}^n^, *ξ*_*i *_are slack variables, and *C *> 0 is the penalty parameter of the error term.

Each data instance in the training set contains the information about the observed phenotypic value as a class label and the SNPs of the subjects as features. The goal of the SVM algorithm is to predict the class label (that is, disease status, "CFS" or "control") of data instances in the testing set, given only the assigned features (that is, the SNP information of the subjects). Given a training set of instance-label pairs, the SVM algorithm maps the training vectors into a higher dimensional space by employing a kernel function and then finds a linear separating hyperplane with the maximal margin in this higher dimensional space. In this study, instance-label training data pairs were used to train an SVM model. Inputs were the SNP genetic markers. Outputs were the CFS status. In our study, we used the following four kernels [21,22]:

• Linear: *K*(*x*_*i*_, *x*_*j*_) = *x*^T^_*i*_*x*_*j*_.

• Polynomial: *K*(*x*_*i*_, *x*_*j*_) = (*γx*^T^_*i *_*x*_*j *_+ *h*)^*d*^, *γ *> 0.

• Sigmoid: *K*(*x*_*i*_, *x*_*j*_) = tanh(*γx*^T^_*i *_*x*_*j *_+ *h*).

• Gaussian radial basis function: *K*(*x*_*i*_, *x*_*j*_) = exp(-*γ*||*x*_*i *_- *x*_*j*_||^2^), *γ *> 0.

Here, WEKA's default settings were used for SVM parameters, that is, *d *= 3, *h *= 0, and *C *= 1. The parameter *γ *was set to 0.05 when we evaluated SVM based on the feature section procedures described in the next section. Otherwise, *γ *was set to 0.01.

Third, the C4.5 algorithm builds decision trees top-down and prunes them using the upper bound of a confidence interval on the re-substitution error [23]. By using the best single feature test, the tree is first constructed by finding the root node (that is, SNP) of the tree that is most discriminative for classifying CFS versus control. The criterion of the best single feature test is the normalized information gain that results from choosing a feature (that is, SNP) to split the data into subsets. The test selects the feature with the highest normalized information gain as the root node. Then, the C4.5 algorithm finds the rest nodes of the tree recursively on the smaller sub-lists of features according to the test. In addition, feature selection is an inherent part of the algorithm for decision trees [24]. When the tree is being built, features are selected one at a time based on information content relative to both the target classes and previous chosen features. This process is similar to ranking of features except that interactions between features are also considered [25]. Here, we used WEKA's default parameters, such as the confidence factor = 0.25 and the minimum number of instances per leaf node = 2.

### Feature Selection

In this work, we employed two feature selection approaches to find a subset of SNPs that maximizes the performance of the prediction model. First, a hybrid approach combines the information-gain method [26] and the chi-squared method [27], which is designed to reduce bias introduced by each of the methods [28]. Each feature was measured and ranked according to its merit in both methods. The measurement of the merit for the two methods is defined as follows. The information-gain method measures the decrease in the entropy of a given feature provided by another feature, and the chi-squared method is based on Pearson chi-squared statistic to measure divergence from the expected distribution. Next, all features were sorted by their average rank across these two methods. After the features were ranked, the classifiers, including naive Bayes, SVM, and C4.5 decision tree, were utilized to add one SNP at a time based on its individual ranking and then select the desired number of the top ranked features that provides the best predictive performance, respectively.

Second, we used the wrapper-based feature selection approach, in which the feature selection algorithm acts as a wrapper around the classification algorithm. The wrapper-based approach conducts best-first search for a good subset using the classification algorithm itself as part of the function for evaluating feature subsets [29]. Best first search starts with empty set of features and searches forward to select possible subsets of features by greedy hill-climbing augmented with a backtracking technique [19]. We applied naive Bayes, SVM, and C4.5 decision tree with the wrapper-based approach, respectively.

### Evaluation of the Predictive Performance

To measure the performance of prediction models, we used the receiver operating characteristic (ROC) methodology and calculated the area under the ROC curve (AUC) [30,31]. The AUC of a classifier can be interpreted as the probability that the classifier will rank a randomly chosen positive example higher than a randomly chosen negative one [31]. Most researchers have now adopted AUC for evaluating predictive ability of classifiers owing to the fact that AUC is a better performance metric than accuracy [31]. In this study, AUC was used as a value to compare the performance of different prediction models on a dataset. The higher was the AUC, the better the learner [32]. In addition, we calculated sensitivity, the proportion of correctly predicted responders of all tested responders, and specificity, the proportion of correctly predicted non-responders of all the tested non-responders.

To investigate the generalization of the prediction models produced by the above algorithms, we utilized the repeated 10-fold cross-validation method [33]. First, the whole dataset was randomly divided into ten distinct parts. Second, the model was trained by nine-tenths of the data and tested by the remaining tenth of data to estimate the predictive performance. Then, the above procedure was repeated nine more times by leaving out a different tenth of data as testing data and different nine-tenths of the data as training data. Finally, the average estimate over all runs was reported by running the above regular 10-fold cross-validation for 100 times with different splits of data. The performance of all models was evaluated both with and without feature selection, using repeated 10-fold cross-validation testing.

## Results

Tables 3, 4 and 5 summarize the results of repeated 10-fold cross-validation experiments by naive Bayes, SVM (with four kernels including linear, polynomial, sigmoid, and Gaussian radial basis function), and C4.5 decision tree using SNPs with and without feature selection. First, we calculated AUC, sensitivity, and specificity for these six predictive models without using the two proposed feature selection approaches. As indicated in Table 3, the average values of AUC for the SVM prediction models of linear, polynomial, sigmoid, and Gaussian radial basis function kernels were 0.55, 0.59, 0.61, and 0.62, respectively. Of all the kernel functions, the Gaussian radial basis function kernel gave better performance than the other three kernels in terms of AUC. Among all six predictive models, the SVM model of the Gaussian radial basis function kernel performed best, outperforming the naive Bayes (AUC = 0.60) and C4.5 decision tree (AUC = 0.50) models in terms of AUC. Moreover, as shown in Table 3, the original C4.5 algorithm without using feature selection approaches used 11 out of 42 SNPs due to the fact that the search for a feature subset with maximal performance is part of the C4.5 algorithm.

**Table 3:** The result of a repeated 10-fold cross-validation experiment using naive Bayes, support vector machine (SVM), and C4.5 decision tree without feature selection.

**Algorithm**	**AUC**	**Sensitivity**	**Specificity**	**Number of SNPs**
Naïve Bayes	0.60 ± 0.17	0.64 ± 0.20	0.52 ± 0.21	42
SVM with linear kernel	0.55 ± 0.14	0.55 ± 0.21	0.56 ± 0.21	42
SVM with polynomial kernel	0.59 ± 0.13	0.46 ± 0.24	0.71 ± 0.21	42
SVM with sigmoid kernel	0.61 ± 0.13	0.62 ± 0.20	0.61 ± 0.19	42
SVM with Gaussian radial basis function kernel	0.62 ± 0.13	0.60 ± 0.20	0.64 ± 0.19	42
C4.5 decision tree	0.50 ± 0.16	0.52 ± 0.21	0.48 ± 0.21	11

AUC = the area under the receiver operating characteristic curve, SNP = single nucleotide polymorphism.

Data are presented as mean ± standard deviation.

**Table 4:** The result of a repeated 10-fold cross-validation experiment using naive Bayes, support vector machine (SVM), and C4.5 decision tree with the hybrid feature selection approach that combines the chi-squared and information-gain methods.

**Algorithm**	**AUC**	**Sensitivity**	**Specificity**	**Number of SNPs**
Naive Bayes	0.70 ± 0.16	0.65 ± 0.21	0.60 ± 0.20	12
SVM with linear kernel	0.67 ± 0.13	0.62 ± 0.20	0.73 ± 0.19	14
SVM with polynomial kernel	0.62 ± 0.13	0.56 ± 0.21	0.68 ± 0.18	9
SVM with sigmoid kernel	0.64 ± 0.13	0.62 ± 0.20	0.67 ± 0.19	4
SVM with Gaussian radial basis function kernel	0.64 ± 0.13	0.58 ± 0.20	0.71 ± 0.18	3
C4.5 decision tree	0.64 ± 0.13	0.80 ± 0.16	0.46 ± 0.20	2

AUC = the area under the receiver operating characteristic curve, SNP = single nucleotide polymorphism.

Data are presented as mean ± standard deviation.

**Table 5:** The result of a repeated 10-fold cross-validation experiment using naive Bayes, support vector machine (SVM), and C4.5 decision tree with the wrapper-based feature selection method.

**Algorithm**	**AUC**	**Sensitivity**	**Specificity**	**Number of SNPs**
Naive Bayes	0.70 ± 0.16	0.64 ± 0.20	0.63 ± 0.19	8
SVM with linear kernel	0.63 ± 0.14	0.71 ± 0.20	0.55 ± 0.21	9
SVM with polynomial kernel	0.63 ± 0.12	0.43 ± 0.20	0.82 ± 0.16	12
SVM with sigmoid kernel	0.64 ± 0.13	0.59 ± 0.21	0.70 ± 0.18	6
SVM with Gaussian radial basis function kernel	0.63 ± 0.13	0.60 ± 0.20	0.66 ± 0.19	7
C4.5 decision tree	0.59 ± 0.16	0.65 ± 0.21	0.55 ± 0.22	6

AUC = the area under the receiver operating characteristic curve, SNP = single nucleotide polymorphism.

Data are presented as mean ± standard deviation.

Next, we applied the naive Bayes, SVM, and C4.5 decision tree classifiers, respectively, with the hybrid feature selection approach that combines the chi-squared and information-gain methods. Table 4 shows the result of a repeated 10-fold cross-validation experiment for the six predictive algorithms with the hybrid approach. As presented in Table 4, the average values of AUC for the SVM prediction models of linear, polynomial, sigmoid, and Gaussian radial basis function kernels were 0.67, 0.62, 0.64, and 0.64, respectively. Of all the kernel functions, the linear kernel performed better than the other three kernels in terms of AUC. In addition, with the hybrid approach, the desired numbers of the top-ranked SNPs for the SVM models of linear, polynomial, sigmoid, and Gaussian radial basis function kernels were 14, 9, 4, and 3 out of 42 SNPs, respectively. Among all six predictive models with the hybrid approach, the naive Bayes (AUC = 0.70) was superior to the SVM and C4.5 decision tree (AUC = 0.64) models in terms of AUC. Moreover, the naive Bayes and C4.5 decision tree algorithms with the hybrid approach selected 12 and 2 out of 42 SNPs, respectively.

Finally, we employed naive Bayes, SVM, and C4.5 decision tree with the wrapper-based feature selection approach, respectively. Table 5 demonstrates the result of a repeated 10-fold cross-validation experiment for the six predictive algorithms with the wrapper-based approach. As shown in Table 5, the average values of AUC for the SVM prediction models of linear, polynomial, sigmoid, and Gaussian radial basis function kernels were 0.63, 0.63, 0.64, and 0.63, respectively. Of all the kernel functions, the sigmoid kernel performed best, outperforming the other three kernels in terms of AUC. Among all six predictive models with the wrapper-based approach, the SVM and C4.5 decision tree (AUC = 0.59) models were outperformed by the naive Bayes model (AUC = 0.70) in terms of AUC. In addition, the numbers of SNPs selected by these six models with the wrapper-based approach were ranged from 6 to 12 SNPs (Table 5). For the naive Bayes model with the wrapper-based approach, only 8 SNPs out of 42 was identified, including rs4646312 (COMT), rs5993882 (COMT), rs2284217 (CRHR2), rs2918419 (NR3C1), rs1866388 (NR3C1), rs6188 (NR3C1), rs12473543 (POMC), and rs1386486 (TPH2).

It is also interesting to compare results between the classifiers with and without feature selection. Feature selection using the hybrid and wrapper-based approaches clearly improved naive Bayes, SVM, and C4.5 decision tree. Overall, both the naive Bayes classifier with the hybrid approach and the naive Bayes classifier with the wrapper-based approach achieved the highest prediction performance (AUC = 0.7) when compared with the other models. Additionally, the use of SNPs for the naive Bayes classifier with the wrapper-based approach (n = 8) was less than the one for the naive Bayes classifier with the hybrid approach (n = 12).

## Discussion

We have compared three classification algorithms including naive Bayes, SVM, and C4.5 decision tree in the presence and absence of feature selection techniques to address the problem of modeling in CFS. Accounting for models is not a trivial task because even a relatively small set of candidate genes results in the large number of possible models [15]. For example, we studied 42 candidate SNPs, and these 42 SNPs yield 2^42 ^possible models. The three classifiers were chosen for comparison because they cover a variety of techniques with different representational models, such as probabilistic models for naive Bayes, regression models for SVM, and decision tree models for the C4.5 algorithm [32]. The proposed procedures can also be implemented using the publicly available software WEKA [19] and thus can be widely used in genomic studies.

In this study, we employed the hybrid feature selection and wrapper-based feature selection approaches to find a subset of SNPs that maximizes the performance of the prediction model, depending on how these methods incorporate the feature selection search with the classification algorithms. Our results showed that the naive Bayes classifier with the wrapper-based approach was superior to the other algorithms we tested in our application, achieving the greatest AUC with the smallest number of SNPs in distinguishing between the CFS patients and controls. In the wrapper-based approach, no knowledge of the classification algorithm is needed for the feature selection process, which finds optimal features by using the classification algorithm as part of the evaluation function [29]. Moreover, the search for a good feature subset is also built into the classifier algorithm in C4.5 decision tree [24]. It is termed an embedded feature selection technique [34]. All these three approaches, including the hybrid, wrapper-based, embedded methods, have the advantage that they include the interaction between feature subset search and the classification model, while both the hybrid and wrapper-based methods may have a risk of over-fitting [34]. Furthermore, SVM is often considered as performing feature selection as an inherent part of the SVM algorithm [25]. However, in our study, we found that adding an extra layer of feature selection on top of both the SVM and C4.5 decision tree algorithms was advantageous in both the hybrid and wrapper-based methods. Additionally, in a pharmacogenomics study, the embedded capacity of the SVM algorithm with recursive feature elimination [34,35] has been utilized to identify a subset of SNPs that was more influential than the others to predict responsiveness to chronic hepatitis C patients of interferon-ribavirin combination treatment [30].

In this work, we used the proposed feature selection approaches to assess CFS-susceptible individuals and found a panel of genetic markers, including COMT, CRHR2, NR3C1, POMC, and TPH2, which were more significant than the others in CFS. Smith and colleagues reported that subjects with CFS were distinguished by MAOA, MAOB, NR3C1, POMC, and TPH2 genes using the traditional allelic tests and haplotype analyses [8]. Moreover, Geortzel and colleagues showed that the COMT, NR3C1, and TPH2 genes were associated with CFS using SVM without feature selection [9]. A study by Lin and Huang also identified significant SNPs in SLC6A4, CRHR1, TH, and NR3C1 genes using a Bayesian variable selection method [14]. In addition, a study by Chung and colleagues has found a possible interaction between NR3C1 and SLC6A4 by using the odds ratio based multifactor dimensionality reduction method [12]. Similarly, another study by Lin and Hsu indicated a potential epistatic interaction between the CRHR1 and NR3C1 genes with a two-stage Bayesian variable selection methodology [13]. These studies utilized the same dataset by the CDC Chronic Fatigue Syndrome Research Group. An interesting finding was that an association of NR3C1 with CFS compared to non-fatigued controls appeared to be consistent across several studies. Thus, this significant association strongly suggests that NR3C1 may be involved in biological mechanisms with CFS. The NR3C1 gene encodes the protein for the glucocorticoid receptor, which is expressed in almost every cell in the body and regulates genes that control a wide variety of functions including the development, energy metabolism, and immune response of the organism [36]. A previous animal study has observed that age increases the expression of the glucocorticoid receptor in neural cells [37], and increases in glucocorticoid receptor expression in human skeletal muscle cells have been suggested to contribute to the etiology of the metabolic syndrome [38]. However, evidence of associations with CFS for other genes was inconsistent in these studies. The potential reason for the discrepancies between the results of this study and those of other studies may be the sample sizes. The studies conducted on small populations may have biased a particular result. Future research with independent replication in large sample sizes is needed to confirm the role of the candidate genes identified in this study.

There were several limitations to this study as follows. Firstly, the small size of the sample does not allow drawing definite conclusions. Secondly, we imputed missing values before comparing algorithms. Thus, we depended on unknown characteristics of the missing data, which could be either missing completely at random or the result of some experimental bias [25]. In future work, large prospective clinical trials are necessary in order to answer whether these candidate genes are reproducibly associated with CFS.

## Conclusion

In this study, we proposed several alternative methods for assessing models in genomic studies of CFS. Our method was also based on the feature selection methods. Our findings suggested that our experiments may provide a plausible way to identify models in CFS. Over the next few years, the results of our studies could be generalized to search SNPs for genetic studies of human disorders and could be utilized to develop molecular diagnostic/prognostic tools. However, application of genomics in routine clinical practice will become a reality after a prospective clinical trial has been conducted to validate genetic markers.

## Competing interests

The authors declare that they have no competing interests.

## Authors' contributions

LCH and SYH participated in the design of the study and coordination. EL performed the statistical analysis and helped to draft the manuscript. All authors read and approved the final manuscript.
